# Community Nurses’ Preparations for and Challenges in Providing Palliative Home Care: A Qualitative Study

**DOI:** 10.3390/ijerph182211838

**Published:** 2021-11-11

**Authors:** Chien-Yi Wu, Yu-Hsuan Wu, Yi-Hui Chang, Min-Shiow Tsay, Hung-Cheng Chen, Hui-Ya Hsieh

**Affiliations:** 1 Department of Family Medicine, Kaohsiung Medical University Hospital, Kaohsiung Medical University, Kaohsiung 80788, Taiwan; dietcokewu0822@gmail.com (C.-Y.W.); yhwu1226@gmail.com (Y.-H.W.); 2 Department of Public Health, College of Health Sciences, Kaohsiung Medical University, Kaohsiung 80788, Taiwan; 3 Department of Nursing, Kaohsiung Medical University Hospital, Kaohsiung Medical University, Kaohsiung 80788, Taiwan; 870140@kmuh.org.tw (Y.-H.C.); minshow0644@gmail.com (M.-S.T.); olive575589@gmail.com (H.-C.C.); 4 Department of Specialist Nurse and Surgical Nurse Practitioner Office, Kaohsiung Medical University Hospital, Kaohsiung Medical University, Kaohsiung 80788, Taiwan

**Keywords:** community nurse, palliative home care, qualitative research, Taiwan

## Abstract

Hospitals have played a leading role in providing palliative care in Taiwan as its care model has developed over the past few decades. However, earlier local studies in Taiwan showed that terminal patients prefer to die at home, highlighting the need to promote community-based palliative care instead of hospital-based care. Along with this shift, how community nurses provide palliative home care merits further exploration. This qualitative descriptive study aims to understand (1) how community nurses implement community-based palliative care, (2) what preparations are needed, and (3) what challenges they may face. Purposive sampling was used for recruiting nurses. We conducted one-on-one, in-depth, semi-structured interviews. Interview recordings were transcribed verbatim and analyzed using thematic analysis. Eight community nurses with a range of experience in palliative home care were interviewed. Four major themes emerged: (1) Opportunities, (2) Qualifications, (3) Support, and (4) Commitments. Psychological preparedness, well-developed professional capabilities, external assistance, and peer support motivate community nurses to offer community-based palliative care. As the requests for palliative home care services increase, community nurses play a critical role in palliative home care. Although the sample size is small and the findings retrieved from a small number of experiences might not be generalized to every region, the study results could inform future experience-sharing and workshop sessions to train more nurses for community-based care, expanding service coverage, and providing optimal palliative care.

## 1. Introduction

The demand for palliative home care is increasing globally, because of both cancer and non-cancer patients’ needs for a more approachable and continuous care system in order to get a rapid response [1,2]. Past researchers have found that palliative home care can relieve symptom burden and improve patient and family satisfaction while reducing the use of and expenditure on medical care resources [3,4,5,6]. A study in Italy found that patients with terminal cancer who receive palliative home care are more likely to die at home, less likely to be hospitalized in the last two months of life, and have relatively shorter hospital stays even when hospitalized [4]. These findings highlight the great potential of palliative home care to improve overall terminal care. Therefore, primary care and community- or even home-based palliative care should be moved higher up in the government’s agenda to enhance palliative care service [1,7].

As people have become more heedful about the quality of end-of-life care for terminal cancer patients and palliative care since the 1990s, Taiwan introduced palliative home care (PHC) as a preferred care model on a trial basis in 1996. Initially, the service covered only terminal cancer patients. Driven by the efforts to bring Taiwan up to par with international standards regarding terminal diseases covered by palliative care, final-stage motor neuron disease, and then eight other categories of non-cancer terminal diseases were included, showing the importance of palliative care to various diseases [8,9]. Over the years, the dominant care model has expanded from home care to palliative care wards, and more recently, to palliative shared care provided by the hospital and professional palliative care teams.

In Taiwan, two categories of palliative home care have been available since 2014. Category A palliative home care is provided by an experienced hospital-based palliative team, while Category B is offered jointly by community general practitioners and nurses. The Taiwan National Health Insurance Administration has stipulated that Category B palliative home care is not an acceptable practice without the assistance of an experienced hospital-based palliative team [10]. According to the data of Taiwan’s National Health Insurance Administration, from January to September 2016, a total of 5971 people received Category A palliative home care, and 716 people received Category B palliative home care. In other words, hospital-based teams provide almost 90% of the palliative home care service, while only a marginal portion of the service is offered by community nurses (CNs) [11,12,13]. However, due to distance, transportation, and human resources constraints, hospital-based palliative home care in many communities is not provided on a real-time, approachable, and continuous basis, resulting in many terminal patients spending their last days in hospitals [13]. In contrast, the World Health Organization highlighted the importance of primary care and community- or even home-based palliative care in developing palliative care [1]. To act on the World Health Organization’s suggestions, Taiwan needs to understand how to deploy and develop community-based palliative home care.

Prior qualitative studies of nurses engaged in palliative home care suggested that relevant preparations and challenges include the individual’s knowledge base, willingness and ambition, and relationship with the patient and his or her family [14,15,16,17]. Externally, awareness of and assistance to inter-team professional care and support from external resources are also critical [16,18]. Remarkably, without sufficient training and preparation in relevant organizations, community nurses may not offer community-based palliative care efficiently. Nevertheless, most of these studies were conducted by western researchers. Asian countries are not sufficiently represented in these studies.

Interestingly, Taiwan data shows that most patients tend to receive hospital-based palliative care service rather than community-based, which is different from western culture. Thus, there is a need to get more in-depth information about palliative home care from nurses currently engaged in community-based palliative care in Taiwan. It is expected that a deeper understanding of local community-based palliative care could inform Taiwan’s plan of meaningfully expanding the coverage of community-based palliative home care.

## 2. Materials and Methods

### 2.1. Design

We conducted a qualitative descriptive study using semi-structured in-depth interviews to examine the following issues: (1) the opportunities and preparations for providing community-based palliative care and (2) the challenges in offering the service. The reporting of this study complies with the consolidated criteria for reporting qualitative research (COREQ) recommendations [19].

### 2.2. Participants

Since community-based palliative home care is not universal, purposive sampling was adopted to find the interviewees efficiently. Community nurses who were officially qualified to provide palliative home care and had at least six months of experience were eligible for the study. We targeted community-based palliative home care nurses assisted by a tertiary hospital in southern Taiwan. Research members informed eligible nurses of the study aims initially by email or message. An interview appointment was scheduled if the nurse agreed to participate.

### 2.3. Data Collection

Researcher CYW conducted a face-to-face semi-structured in-depth interview with each participant at a quiet place of the participant’s preference (e.g., workforce, café) from 21 May 2020 to 14 May 2021. Researcher Y.H.W. presented in the interviews as an observer assisting in audio recording. Researcher C.Y.W. is a male palliative care physician with 14 years of clinical experience, 10 years of palliative care experience, and a Ph.D. student who has undergone qualitative courses. Y.H.W. is a female intensive care unit registered nurse and Master’s student in nursing who has undergone qualitative courses.

Eight community nurses were asked and interviewed. No participants refused or dropped out of the study. Since all participants were assisted by the palliative team from the southern territory hospital where C.Y.W. works, they have some rapport with researcher C.Y.W. Each interview generally lasts between 45–70 min. Interviews were audio-recorded, and field notes were taken after each interview by C.Y.W. and Y.H.W. to reflect any issues from interviewees’ responses during interviews. The interview guide was developed based on literature reviews (see Table 1). Follow-up questions were asked based on the responses of the participants. The research team stopped seeking new participants to interview at data saturation. Interview records were then transcribed verbatim. Transcripts were not returned to participants for comments or correction, and no repeated interviews were conducted.

### 2.4. Analysis

Subsequently, the qualitative interview data were analyzed by thematic analysis. Manual techniques and analysis software MAXQDA (version 12.0.3) were used to organize text fragments and coding. The analysis involved transcribing data, repeated reading, noting meaningful concepts into codes, grouping codes into themes, and reviewing and renaming themes [20]. For example, the statement “After we care for many cases like this, I feel a little bit of a sense of mission. With our help, they (patients) can be more confident about home care and dying at home” was coded under the subthemes “Accompany, peaceful death, and farewell“. This subtheme was later merged with other related subthemes such as “Hard to let go—the will to do good ”and “Be on call twenty-four seven” into the theme “Commitments.” Consensus discussions with the research team, including C.Y.W., Y.H.W., and H.Y.H. were carried out during coding and interpretation to avoid bias blind spots. Researcher H.Y.H. is a female palliative nurse practitioner with 28 years of clinical experience, 17 years of palliative care experience, and a Master’s degree in nursing. She has plenty of experience in qualitative studies.

### 2.5. Ethical Consideration

The study was conducted following the Declaration of Helsinki and approved by the Institutional Review Board of Kaohsiung Medical University Hospital (KMU-HIRB-E(I)-2020010). Written informed consent was obtained before the start of the interview. The participants were well informed about their rights to withdraw from the study at any time without explanation. The interview records were well-kept with locked passwords on a computer, and we assured anonymity and confidentiality in every process. We confirmed that the data would be deleted after three years.

## 3. Results

**Figure 1:**
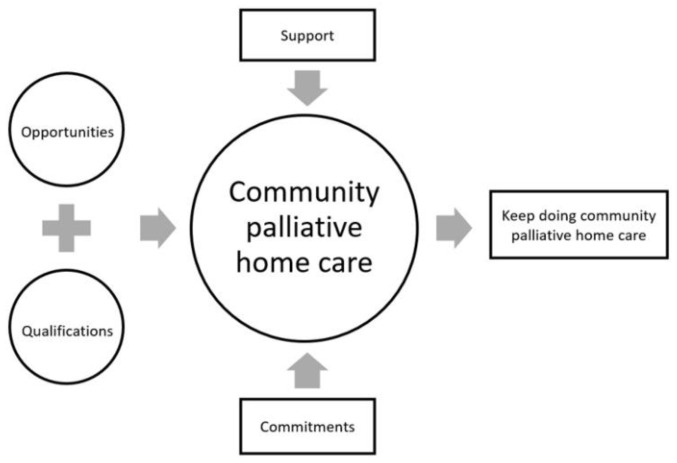
Themes from the study.

Table 2 presents the data on the participants. After analysis, four themes and twelve subthemes were identified (see Figure 1): (1) Opportunities; (2) Qualifications; (3) Support; and (4) Commitments.

### 3.1. Opportunities

Several participants mentioned that they engaged in palliative home care mainly because a good opportunity arose for which they happened to be qualified.

#### 3.1.1. Transition to Community Palliative Nursing

The participants gained experience after working in their original jobs for some time. They changed course when a good opportunity arose. They started businesses, established partnerships with their friends, or were employed by home care practices as nurses to provide community-based palliative home care:

*“By coincidence, my husband said: ‘Would you like to quit your job and run your own business? You have so many ideas. Do you want to start a business of your own and do what you like to do?’” (A)*

*“I have been working as a nurse for 23 years. I was transferred to a home care practice for training. Only when engaged in, did I learn what home care truly is; I could observe the whole home care processes very closely and carefully.” (B)*

*“I have been working at the care center since I left the hospital. Then, I started home care practices due to our business scope. At that time, I thought it was okay to provide home care because I keep in touch with the elders I knew from the care center. So, if I could enter their houses, I could see the whole picture of home care.” (F)*

*“I have been working in the hospital; after two years of clinical experience, I had a plan for my future because of the broad field of nursing. Well, I thought long-term care is the trend after I doing some researches. I also sense the increasing needs for home-based care. Then I started adult daycare service and home care practices.” (G)*

The participants did not decide to provide home care based on instinct. They committed to home care because they had accumulated work experience and had a plan for career development before a suitable opportunity arose.

#### 3.1.2. Follow Your Heart—The Passion for Palliative Care

Many participants mentioned that they had experience with palliative care while working in hospitals. They realized that this was what they like. In other words, they already had the passion and the desire to work in palliative care.

*“I used to work in the intensive care unit, and I had seen many patients with lung cancer, and I thought, they are so young… Do they have to be intubated? Must they suffer from these treatments? At that time, I noticed I had a sense of destination for palliative care.” (A)*

*“To be honest, I have been trying to quit palliative care, but it seems that someone has been pushing me, telling me that I must go back to what I want to do originally, and to face it.” (A)*

### 3.2. Qualifications

As a community nurse in palliative home care, one must face many challenges and unexpected situations on one’s own and adapt accordingly to meet the needs of patients and their families. In addition to comprehensive knowledge and skills, a confident attitude is also necessary to successfully serve patients and their families.

#### 3.2.1. Confidence Is Not to Look Down on Yourself

Many community nurses thought that providing community-based palliative home care means more than their familiar nursing services. Training courses in long-term care equipped participants with abilities related to essential home-based rehabilitation or nutritional assessment and advice. Therefore, they believed that the service scope should not be confined to nursing. They should be confident in their abilities and provide primary inter-disciplinary care to home-based patients, thus offering a better service to their patients.

*“With my professional ability, I am confident to make patients feel better; I am confident because I went through training, and I do not think we should underestimate ourselves.” (A)*

*“I think home care is my work of interest, and then I thought what else I could do, such as long-term care 2.0? After training in long-term care, I think we nurses should not underestimate ourselves due to our expertise.” (A)*

#### 3.2.2. Responsiveness from Training in Hospital Routines

In palliative home care, unlike with hospital patients whose problems are mainly related to the admitted department, nurses often encounter various problems. In home-care settings, palliative home care nurses are first-line contacts for patients and their families. They should address, note, and summarize complicated issues that patients and their families have raised and then give primary responses. Although it is possible to seek assistance from community physicians or even professional teams of the backup hospital afterward, first-line responsiveness is critical. Fortunately, participants had such abilities, developed through previous hospital working experience.

*“If I had not worked in the hospital first, maybe I would have found the job of home care particularly challenging. Because of sufficient training in the hospital, I am well prepared for palliative home care. A crisis can happen anytime in palliative home care, and family members may raise various questions, including caring for colostomy and inserting tubes. For home care service, you need to care for patients with multiple diseases such as CKD, DM, stroke, etc. If I am also providing palliative care, there are also palliative care problems to be solved. In other words, only when you are well prepared can you cope with such a situation…” (B)*

#### 3.2.3. Regarding Skills, Practice Is More Important Than Training Courses

So far, physicians and nurses who intend to offer community-based palliative home care must receive education and training in advance. Although the requirement is justifiable, in practice, education and training alone cannot fully equip community nurses to handle complicated and volatile home care issues that may arise in terminal patients, nor provide community nurses the confidence to face and address issues related to palliative patients. With an experienced hospital-based palliative care team’s assistance, community nurses have more confidence in dealing with complex issues during clinical practices.

*“I think practice is more important than courses because if I had not gone to the hospice ward and done the job for a few months… I would be terrified when caring palliative patients” “(After practice) You will know what problems we might encounter in the process and to know what the family might need.” (B)*

*“Palliative course used to be incomprehensible. After practice (palliative care), you will understand it completely.” (C)*

*“It is easy to forget the things from the class. But once you start (palliative practice), you will remember what you have learned.” (C)*

### 3.3. Support

Participants also mentioned the importance of hospital backup and peer support when they engaged in community-based palliative home care. With support, they felt more confident in implementing the service.

#### 3.3.1. Hospital Backup

Participants reported feeling more confident in caring for terminal patients in their communities when supported by the hospital’s experienced palliative team, since the problems of terminal patients are often complex and diverse. When community nurses face issues that they cannot control or deal with, the palliative team from the backup hospital can give them treatment suggestions, and if needed, the patient can be transferred to the hospital for specific care in the palliative ward. With the hospital backup, community nurses feel at ease when engaging in community-based palliative care.

*“At first, I had many thoughts, but I did not know how to conduct… With the support from the hospital team, I felt safe and realized that I could ask for backup whenever facing difficulties.” (A)*

*“The current way that the hospital cooperates with us is what I wished to have when I started home care service. When I could not handle the situation, the team from the hospital can take care of them…” (D)*

#### 3.3.2. Peer Support

In addition to the support of the backup hospital, participants also mentioned peer support. Participants reported that they relieved mental pressure by discussing patients’ difficulties and sharing grievances with peers while working at the hospital. Since the community nurses work independently, the backup hospital set up an online communication network that enables community nurses to get to know each other, share clinical distress, and advise one another.

*“When working in the hospital, you do feel less stressed since you have so many peers to chat or discuss with. Thankfully, with the online peer network, we can discuss any difficulties we encountered among the cases.” (B)*

*“When doing home care service, you can get to know more people, likely from another home care setting. Everyone has their way of nursing practice, so we will discuss our cases, seeking for a different point of view.” (E)*

### 3.4. Commitments

There are many challenges that those engaging in community-based palliative home care must overcome and commitments to what they think of as the right thing. For many participants, their commitments to home care of terminal patients are mainly motivated by their sense of mission.

#### 3.4.1. Hard to Let Go—The Will to Do Good

While engaging in community-based palliative home care, all the participants are aware of the difficulties entailed by the job. Although palliative home care is hard work, it is still meaningful, especially in helping patients die peacefully and supporting family members. Participants consider the work rewarding, and thus found it difficult to give up palliative home care jobs.

*“Being a home care nurse, you have to be optimistic and enthusiastic about your work… you do extra work that is meaningful to the family without paid… but you are still willing to help them.” (C)*

*“It (home care) is valuable… not about money… but to help your patient…” (F)*

*“If I had to leave the home care service, I would miss the palliative part most because palliative care is what everyone needs no matter what. Whom is there to help through the end of life is quite important.” (F)*

*“If you want to do palliative care, you need mental preparation. Because it is definitely not easy.” (F)*

#### 3.4.2. Be on Call Twenty-Four Seven

Home care differs from hospital care due to the need to be prepared to receive calls from patients or family members, because when terminal patients are at home, the first line of assistance for patients and their families in case of emergencies is often the community nurses. Therefore, community nurses should always be prepared and committed to deal with patients’ acute situations so that patients and their families can feel assured at home.

*“You will get their LINE message, their phone call at any time, even on holiday nights… They may keep asking you about trivial things.” (F)*

*“If you want to offer palliative care, you should forget about the difference between on- and off-duty. Once you treat the patient as your family member, the patient and family members will trust and rely on you… then I accompany them until the end of life, which is the most meaningful thing.” (F)*

*“In palliative care, you should always answer phone calls! That is, you could not get a good rest during private time. Because sometimes you will get calls in the early morning. Calls also come in during the night.” (G)*

#### 3.4.3. The Process of Building a Relationship Implies That You Should Always Be There

When building a relationship between home care providers and patients and family members, it is crucial to let the patients and family members know that home care nurses are always there to help.

*“After several visits and problems solved, we get closer and have such a relationship established.” “Patients will know you are always there for them.” (F)*

#### 3.4.4. Accompany, Peaceful Death, and Farewell

To many community nurses, palliative home care means physical presence in the patient’s house. Compared with the work model in hospitals, the nurse could spend more time caring and talking to patients and their family members. During such processes, the nurse could demonstrate the value of constant companionship. The most important thing is to accompany the end-of-life patient until their peaceful death. The participants reflected that such a process is for them a memorable experience:

*“After we care for many cases like this, I feel a little bit of sense of mission. With our help, they (patients) can be more confident about home care and to dying at home.” (B)*

*“I think it is essential to spend time sitting down and talk… listen to the memories about her husband, about her dad… it is more about accompany.” (B)*

*“So sometimes they do not give you much oral feedback. Once he or she died peacefully, I feel mission completed!” (F)*

#### 3.4.5. Trust and Dependence Is the Key to Motivation

Participants felt that they had gained the trust of terminal patients and their family members while caring. The patient and the family members would rely on home care nurses when facing problems seeking suggestions and assistance. The trust and dependence from the patient and family members motivate community nurses to keep working in palliative home care.

*“I think it is because he trusts in you so that you will treat them as your own family.” (B)*

*“When the family members see your professionalism, you are recognized. Then, the family members will follow your instructions to take care of the patient. You will be happy to see the achievement.” (C)*

*“The family members and patient believe in me and rely on me thus, and they will let me support them not only physically but spiritually. Once they believe in you, they will tell you some personal issues that you might not have been able to ask before, that is, very meaningful.” (F)*

*“Some patients are still very dependent… on the medical center. Yeah, and they don’t trust us deeply. Well, there are still many people who give us the sense of trust, open to receive our care.” “Sometimes, they contact us even after the patient’s funeral… thanking us for the support… which is very positive and delightful.” (G)*

## 4. Discussion

### 4.1. Main Findings

From the results of our study regarding the preparations and challenges of community nurses for engaging in community-based palliative home care, four themes were identified. First, the nurses should be willing to change their career track and be sufficiently equipped with qualifications from prior nursing experiences. In the actual implementation process, peer support and the availability of a hospital-based palliative team are important. Finally, when they devote themselves to palliative home care, they could sense the value and importance of work, motivating them to commit to and implement palliative home care.

Regarding the decision to commit to community-based palliative care, the results show that accumulating clinical experiences in the past strengthens their determination. Plans for career development or the invitation of colleagues or friends also played a role in persuading the participants to give up their original jobs and devote themselves to community-based palliative home care. Similar dynamics were also identified in prior qualitative studies [15,21,22]. While experiencing their work, nurses would be led to reflect on the value of the job as something they are determined to do and then devote themselves to palliative home care. In other words, internal motivation is an essential factor that leads to commitment in community-based palliative care.

Relevant training and education before engaging in palliative home care were highlighted by research [14,16,18,23,24]. Some studies have argued that education and training could enhance community nurses’ ability to provide palliative care [23]. However, our study shows that education and training alone are not sufficient. Actual practices are more critical, as they offer the opportunity to employ the knowledge acquired for mutual verification, making education and training more valuable and enhancing community nurses’ confidence in caring for terminal patients. The results corroborate the findings of Stajduhar in 2011, who stressed the critical role of combining professional skills with desirable practices in implementing home care [25].

Participants reported the importance of responsiveness. In palliative home care settings, various clinical care needs and the challenges of communicating with family members are rather demanding. The participants felt that the foundation of hospital routines is valuable, equipping them with sufficient capabilities to cope with these issues. This finding is confirmed by prior studies, which indicated that implementing community-based palliative care requires coping with complex and variable situations [18,22]. Moreover, participants said that community nurses should be comfortable providing comprehensive services for community-based palliative home care. Studies have suggested that community nurses believe that they could provide such high-quality care and inter-disciplinary care [16,18,26].

Regarding “Support,” it is essential for sustained community-based palliative care. The support system includes assistance from the backup hospital and the community-based nurses’ peer support network. The support system was also mentioned in the literature [18,27,28]. After all, palliative care is an interdisciplinary effort. For complex challenges, assistance from peers or a backup hospital’s palliative team is imperative, possibly providing more emotional support, enhancing coordination, and improving communication to promote the living quality of patients and their families—thereby encouraging the community-based palliative team to devote themselves to palliative home care continuously.

Finally, regarding “commitment,” the results suggest that it complements the above categories. It is also mentioned frequently in the literature as an essential aspect of palliative home care [14,15,21,26,28,29]. Professional capacity and the willingness to engage in community-based palliative care are the most important. With professional care, the needs of patients and their families can be met. Moreover, an excellent relationship and mutual trust provide the client with the confidence to assist the terminal patient to live through the end of life peacefully. Meanwhile, nurses felt satisfied when the client recognized their professional care services. This recognition enhanced community nurses’ self-identity and nursing values. Therefore, the participants were more willing to work hard as community-based palliative care providers and felt that their service was significant.

### 4.2. Study Limitations

There are limitations to this study. First, the participants are mainly nurses from home care practices assisted by the researchers’ hospital. The results might differ if community nurses from other regions were covered. The sample size is small, and the findings were retrieved from a small number of experiences which might differ from other areas, and thus cannot be generalized. Therefore, future studies could increase generalizability by considering questionnaire surveys in other regions. Second, given Taiwan’s national health insurance system, the nursing payment for community-based palliative home care is lower than general home care. As more experienced community nurses may shy away from community-based palliative home care due to income, this might compromise the completeness of our data. However, from another point of view, the participants in our study are highly prepared and enthusiastic about palliative home care in the community; we may thus better understand how to prepare for working in community-based palliative home care by studying them.

## 5. Conclusions

Preparations are needed for implementing community-based palliative home care, covering education and training and partnerships with peers and support from the backup hospital. Only through having such support could community nurses cope with distresses and difficulties successfully. However, nurses’ experiences and beliefs are also essential. They affect nurses’ willingness to engage in community-based palliative care. Therefore, for the sustainable development of community-based palliative care, internal factors also play a significant role. Again, the factors are related to community nurses’ clinical experiences and personality traits. A multi-dimensional approach is needed through academic workshops and relevant, continuing education to gradually enhance nurses’ willingness to engage in community-based palliative care. If supported by a willing community-based palliative care team and a backup hospital network, we are confident that community-based palliative care could be prompted.

## Figures and Tables

**Table 1:** Semi-structured interview guide.

Questions
1. How did you get involved in the home care nursing field?
2. Why did you engage in palliative home care?
3. What supports you to continue working in the palliative home care field?
4. What does palliative home care mean to you?

**Table 2:** Demographic Characteristics of Home Care Nurse Participants.

Codename	Gender	Experience in Acute Nursing (Year)	Experience in Community Nursing (Year)	Experience in Palliative Home Care (Year)	Interview Time (Min)
A	F	23	3.5	2.5	61
B	F	25	3	2	65
C	F	31	18	4	68
D	F	19	5	5	45
E	F	20	4	2.5	45
F	F	20	2	2	60
G	M	6	4	1.5	45
H	F	24	4.5	1	49

## Data Availability

The data presented in this study are available on request from the corresponding author. The data are not publicly available due to the conditions specified in the data protection contract for this study.
